# The Protective Effects of Sufentanil Pretreatment on Rat Brains under the State of Cardiopulmonary Bypass 

**Published:** 2015

**Authors:** Kun Zhang, Man Li, Xiao-chun Peng, Li-shen Wang, Ai-ping Dong, Shu-wei Shen, Rong Wang

**Affiliations:** a *Department of Anesthesiology, Jingzhou Clinical Medical College, Yangtze University, Jingzhou, Hubei Province,. China.*; b *Department of Oncology, Jingzhou Clinical Medical College, Yangtze University, Jingzhou, Hubei Province, China.*; c *Department of Pathophysiology, Medical School of Yangtze University,Jingzhou, Hubei Province, China.*

**Keywords:** Sufentanil, Pretreatment, CPB, Total calcium in brain tissue, S100β

## Abstract

This study aimed to observe the protective effects of sufentanil pretreatment on rat cerebral injury during cardiopulmonary bypass (CPB) and to explore the underlying mechanism. Twenty-four male adult Sprague Dawley (SD) rats were divided into 4 groups. Then, the rat CPB model was established. A 14G trocar was inserted into the atrium dextrum. For rats in S1 and S5 groups, sufentanil (1 µgKg^-1^ and 5 µgKg^-1^) were applied before CPB process. After the operation, rat brain samples were harvested for measurement of the water content of the brains, total calcium in brain tissue and the level of serum S100β. Compared with the Sham group, the water content and the total calcium of the brain tissue, and the expression of S100β in serum were significantly increased in the CPB group (*P*<0.05). Compared with the CPB group, sufentanil treatment significantly reduced the water content of the brains, the total calcium and S100β expression (*P*<0.05). The blood pressure and heart rate were significantly decreased in groups CPB, S1, and S5 compared with Sham group during CPB. Compared with the Sham group, the levels of pH and blood lactate in other groups were decreased and increased, respectively, in the post-CPB period. During the CPB and post-CPB periods, the hematocrit levels were significantly down-regulated in groups CPB, S1, and S5 compared with Sham group. In conclusion, sufentanil pretreatment was effective in reducing the cerebral injury during CPB. Reduction in calcium overload may be a potential mechanism in such process.

## Introduction

During the period of cardiopulmonary bypass (CPB), cerebrovascular microemboli (including deciduous aortal atheromatous plaque, aeroembolism and tissue fragment), brain metabolic disturbance and inflammatory factors, are the main causes of cerebral injury, which lead to a series of complications after CPB operation(1, 2). The CPB affects multiple aspects in human, such as CBF-blood pressure autoregulation, pH, and blood lactate (Lac) *etc* (3, 4). Cognitive impairment was a major neurological complication during the perioperative period of CPB (5). Previous studies showed that children with surgery or CPB for the treatment of ventricular septal defect, suffered from further cerebral dysfunction (6). Previously, animal studies have also showed that the decreased cognitive function during the perioperative period of CPB is correlated with cerebral injuries (7). 

S100β protein is a kind of neuropeptide, which is highly expressed in blood serum when the brain suffering from severe cerebral injury (8, 9). Most brains with cerebral injuries are accompanied with severe encephaledema (10). So by examining the expression level of S100β and the brain water content can evaluate the severity of cerebral injury (11). Moreover, it has been reported that calcium overload is one of the potential mechanisms of cerebral injury (12). 

Sufentanil is the most potent of the synthetic opioids and is a new-type μ opiate receptor stimulant (13, 14). Opioids have been used to achieve cardiac anesthesia in clinic (15). During CPB cardiac surgeries, sufentanil was mainly used for anesthesia, while recently it has been proposed to be effective in preventing cerebral ischemia and hypoxia injury (16). Human studies have shown that pharmacokinetics of sufentanil can be changed during CPB (17). Thus the concentration of sufentanil changes in association with the process of CPB (18). However, few studies were done on the protective effects of sufentanil pre-administration on cerebral injury in CPB cardiac surgeries. Here in the present study, we studied the protective effects of the sufentanil pretreatment on cerebral injury of rats by examining the brain water content and total calcium concentration, the blood serum S100β, the blood pressure, the heart rate, and the blood gas analysis rat brains in each condition*. *This investigation will provide valuable tools to mitigate cerebral injury causes during CPB. 

## Experimental

*Experimental materials*

Animals: Twenty-four healthy male Sprague Dawley (SD) rats with an average weight of 400±20 g, were provided by the Animal Experiment Center, Medical College, Yangtze University. The rats can get access to food and water *ad libitum*. All the experiments were approved by the animal control committee.

*Experimental grouping and method of administration*

Rats were divided into 4 groups randomly, including Sham CPB group, CPB group and groups of the sufentanil (Batch number, 12101934, Yichang Humanwell Pharmaceutical Co., *Ltd*., Hubei Province) pretreatment with different doses (S1 and S5). The Sham group had the same operations except that CPB was not performed. Before the CPB process, loading doses of sufentanil (1 µgKg^-1^ and 5 µgKg^-1^) were administrated to rats in the S1 and S5 groups. 

*Anesthesia*

Before the anesthesia, rats were injected with 0.02 mgKg^-1^ of atropine as pre-anesthetic. For anesthesia, 4% - 6% of isoflurane was used for induction of anesthesia and then 2% was used to keep the anesthesia status. After the anesthesia, trachea cannula was performed through the mouth as well as the mechanical ventilation at the frequency of 60 times per minute, and 0.1 mgKg^-1^ of vecuronium bromide was injected intraperitoneally.

*Construction of CPB models*

**Figure 1:**
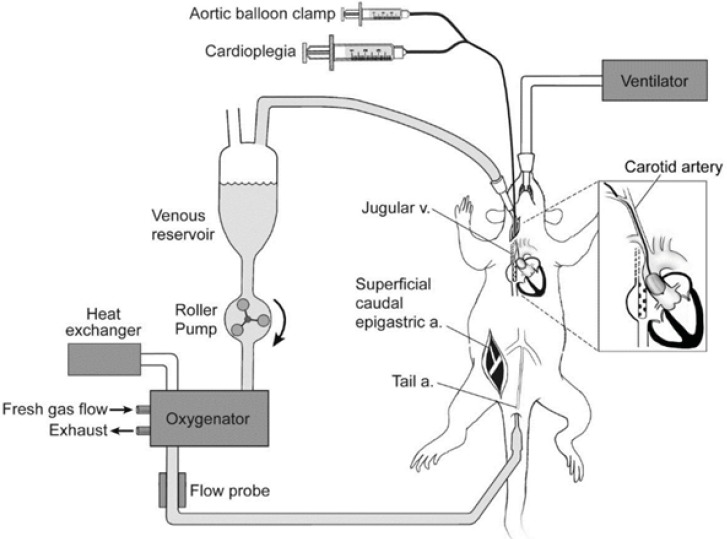
CPB models of rat (www.cardiothoracicsurgery.org).

CPB models of rats were made according to an altered CPB model established by Mackensen G (19) (Figure 1). Briefly, after heparinization (500 µgKg^-1^), arterial pressure was monitored, and arterial blood gas was analyzed. 20G trocars were placed at caudal artery as the ends of CPB infusion. A 14G trocar was inserted in the atrium dextrum. The junction at the post-cava was regarded as the exit of CPB, so that the drainage was sufficiently carried out as there was a 40 cm fall.

CPB loop was composed of blood container, constant-flow peristaltic pump (Mosterflex standard digital drive pump, Cole-Parmer Instrument Co., USA), and micro oxygenator for rats with an effective oxygenation area of 0.09 m^2^ (Fudan Biological Material Co., *Ltd*., Shanghai) and connecting pipes. Twenty milliliters no-blood fluid was prepared for CPB, including 12 mL lactated Ringer's fluid, 7 mL 6% hydroxyethyl starch and 1 mL mannitol. During CPB, crystal glue solution was added to the blood container to keep the quantity of blood at 2-3 mL in the container. 

The initial perfusion rate of CPB was 100 mLKg^-1^·min^-1^, and then 160 mLKg^-1^·min^-1^ with an oxygen flow rate at 0.2-0.5 L/min. The rectal temperature was 26-28 °C, and 20 minutes before ending of CPB, 42 °C water was used to re-warm the animal until the rectal temperature reached 36 °C. The total duration of CPB was 1.5 hours, and 2% isoflurane was infused to keep the depth of anesthesia. After CPB, the breath and circulatory stability was kept for one more hour.

*Measurement of water content and total calcium in brain and S100β in serum*

Rats were decapitated and the brains were removed rapidly. The wet weight was measured by an electronic balance. Then, the brains were placed into a drying oven at 60 °C for 72 hours. The dry weight was measured and the water content of the brain was calculated according to the following formula: the water content of the brain = (wet weight – dry weight) / wet weight ×100%. 

One hour after the operation, 3 mL venous blood was extracted and centrifuged (4000 r·min^-1^ for 10 min), and the blood serum was collected into 1.5 mL Eppendorf (EP) pipes. S100β-ELISAkit (96t, GBD Company, American) was used to measure the content of the blood serum S100β according to the manufacturer instructions. 

Rat forebrains were mixed with 4 mL mixture of nitric acid and perchloric acid (4:1 in volume) overnight. The total calcium content in brain tissue was measured using an inductively coupled plasma-optical emission spectrometer (ICP-OES) model Vista PRO from Varian (Victoria, Australia).

*Measurement of blood pressure, heart rate, and blood gases*

The blood pressure and heart rate were recorded at 5 min before CPB, every thirty minutes during CPB period, and at 30 min and 60 min after CPB period. Measurement techniques for the blood pressure and heart rate were as described by Plehm *et al*. (20). The arterial blood gas analyses were measured at 5 min before CPB, at 60 min during CPB, and at 30 min and 60 min after CPB period according to Wang *et al*. (21).

*Statistical method*

All data were analyzed using SPSS11.5 software. Results were represented as Mean± standard deviation (SD). One-way ANOVA and post-hoc turkey test were used for comparison among groups. *P*<0.05 was considered as statistical significance.

## Results

*The effects of sufentanil pretreatment on the water content of brain during the period of CPB*

Compared with the Sham group, the water content of brain in the CPB rats was significantly increased (*P*<0.05). Compared with the CPB group, both sufentanil pretreatment groups significantly decreased the water content of rat brains (*P*<0.05), so that sufentanil pretreatment has protective effect on brain injuries caused by CPB (Table 1). 

*The effects of sufentanil pretreatment on the total calcium level of rat brain during* *the period of CPB* *.*

Compared with the Sham group, the total calcium was significantly increased in rats in the CPB group (*P*<0.05). Compared with the CPB group, sufentanil pretreatment significantly reduced the total calcium level in the brain tissue of rats in both S1 and S5 groups during CPB cardiac surgeries (*P*<0.05) (Table 1). 

**Table 1:** The water content in brains, the total calcium in brain tissues and the expression of serum S100β in the four groups of rats.

**Groups**	**The dose of sufentanil** µgKg^-1^	**The water content in brain** %	**S100β expression** Pg mL^-1^	**The total calcium in brain** µg g^-1^
Sham	－	78.15±1.61	309.55±36.24	64.03±13.19
CPB	－	86.12±2.49*	561.03±71.28*	112.86±11.76*
S1	1	80.03±1.74△	429.62±45.89*△	77.00±13.26*△
S5	5	82.36±1.53*△#	452.66±39.67*△	83.9±10.32*△

The values are presented as Mean±SD (n=6).

* *P*<0.05, compared with the Sham group;

△ *P*<0.05, compared with the CPB group;

# *P*<0.05, compared with the S1 group.

*The effects of sufentanil pretreatment on the expression of serum S100β during the period of CPB*

Compared with the Sham group, serum S100β in the CPB group was significantly increased (*P*<0.05). Compared with the CPB group, sufentanil pretreatment significantly decreased the expression level of S100β in both S1 and S5 groups (*P*<0.05), while the S100β was still higher than that of the Sham group (*P*<0.05) (Table 1).

*Influence of sufentanil pretreatment on* *blood pressure and heart rate* *of CPB*

The blood pressure and heart rate were detected before, during, and after the CPB to investigate whether they were influenced by pretreatment with *sufentanil* (Table 2). The results showed that the blood pressure decreased significantly during CPB period and then recovered at 60 min in the post-CPB period of group CPB, S1, and S5. The heart rate also decreased in groups CPB, S1, and S5 during CPB surgery, and then recovered to base level at 90 min in post-CPB period. However, there were no significant differences in blood pressure and heart rate in sufentanil pretreated groups, S1 and S5, compared with CPB. 

**Table 2:** The blood pressure and heart rate analysis in the periods of pre-CPB, CPB, and post-CPB. Two indexes were measured at 5 min in pre-CPB; at 30 min, 60 min, and 90 min during CPB; at 30 min and 60 min in post-CPB. In this table, MAP means arterial blood pressure and HR means heart rate.

**Index**	**Group**	**Pre-CPB 5 min**	**CPB 30 min**	**CPB 60 min**	**CPB 90 min**	**Post-CPB 30 min**	**Post-CPB 60 min**
	Sham	95.3±12.1	96.9±10.8	100.5±12.2	103.4±12.6	97.8±9.0	98.0±10.9
MAP	CPB	99.7±15.2	69.8±11.4**	70.1±9.1**	71.9±6.6**	88.5±8.4*	91.3±9.9
(mmHg)	S1	101.4±12.7	73.3±8.8**	72.6±7.6**	73.0±7.9**	89.4±8.5*	94.1±11.1
	S5	97.6±12.5	72.2±9.4**	71.8±8.5**	70.7±7.4**	90.2±7.6*	95.21±10.5
	Sham	249.8±41.9	236.1±44.1	230±47.2	231.3±43.3	233.8±46.8	228.1±45.5
HR	CPB	237.1±34.2	211.9±39.4	198.6±38.6	201.4±43.8*	209.8±37.2	212.0±33.6
(bpm)	S1	238.5±46.8	189.3±36.7*	185.3±41.4*	195.3±44.2*	194.9±39.2*	202.7±38.9
	S5	233.7±44.1	182.6±44.7*	176.1±42.8*	187.1±47.5*	186.5±35.6*	199.5±42.3

The values are presented as Mean±SD (n=6).

* *P*<0.05 and

** *P*<0.01, compared with the Sham group.

*Blood gas analysis* *in the periods of* *pre-CPB, CPB, and post-CPB*

Other parameters of blood gases including pH, arterial oxygenation (PaO_2_), arterial carbon dioxide partial pressure (PaCO_2_), Lac, and hematocrit (Hct) were analyzed to further investigate the influence of sufentanil pretreatment on rat in the perioperative period of CPB (Table 3). The results showed that there were no significant differences in PaO_2_ and PaCO_2_ during the perioperative period of CPB in groups CPB, S1, and S5 compared with Sham group. The pH values were decreased significantly in groups CPB, S1, and S5 in comparison with Sham group at 30 min and 60 min after CPB treatment. The levels of Lac were elevated significantly at 30 min and 60 min during post-CPB in groups CPB, S1, and S5 compared with Sham group. Sham group had the lowest Hct contents among all groups during CPB and post-CPB. However, there were no statistically significant differences in the levels of pH, PaO_2, _PaCO_2, _Lac, and Hct, respectively, ingroups S1 and S5 compared with CPB during pre-CPB, CPB, and post-CPB periods.

**Table 3:** Arterial blood gas analysis in pre-CPB, CPB, and post-CPB. Arterial blood gas including pH, PaO_2, _PaCO_2, _Lac, and Hct were measured at 5 min before CPB, at 60 min in CPB, and at 30 min and 60 min in post-CPB, respectively. In this table, PaO_2_meansarterial oxygenation; PaCO_2_means carbon-dioxide tension in arterial blood; Lac means locate; Hct means henatokrit.

**Index**	**Group**	**Pre-CPB 5 min**	**CPB 60 min**	**Post-** **CPB 30 min**	**Post-CPB 60 min**
	Sham	7.416±0.050	7.414±0.048	7.413±0.045	7.418±0.042
	CPB	7.404±0.049	7.386±0.033	7.335±0.019**	7.380±0.017*
pH	S1	7.419±0.047	7.396±0.040	7.366±0.023**	7.371±0.026*
	S5	7.411±0.042	7.389±0.044	7.363±0.021**	7.378±0.029*
	Sham	357.6±30.3	359.4±32.1	358.5±31.4	328.5±34.0
PaO_2_	CPB	362.1±28.1	374.5±30.1	356.5±25.9	281.5±29.1
(mmHg)	S1	357.3±30.7	369.1±28.5	314.1±24.2	306.0±22.2
	S5	355.3±29.2	364.6±27.1	316.7±22.9	308.0±24.1
	Sham	39.6±4.2	39.4±3.7	39.1±4.0	39.3±3.8
PaCO_2_	CPB	41.4±4.1	38.6±3.7	38.9±2.6	38.6±2.6
(mmHg)	S1	40.6±4.2	39.8±3.0	38.0±2.5	37.6±3.0
	S5	40.2±4.3	39.5±3.2	38.5±2.8	37.1±3.5
	Sham	4.09±0.31	4.05±0.34	4.07±0.32	4.06±0.33
Lac	CPB	4.13±0.36	4.45±0.38	6.21±0.80**	5.33±0.51*
(mmol·l^-1^)	S1	4.06±0.39	4.41±0.35	6.09±0.41**	5.24±0.53*
	S5	4.08±0.35	4.42±0.37	6.11±0.43**	5.27±0.55*
	Sham	42.74±5.17	41.44±4.65	38.63±3.82	36.91±3.86
Hct	CPB	41.16±4.62	25.93±1.93**	26.13±1.84**	26.14±1.77**
(%)	S1	41.51±4.75	26.34±1.67**	26.69±1.43**	26.98±1.63**
	S5	41.48±4.69	26.42±1.59**	26.87±1.66**	26.54±1.71**

The values are presented as Mean±SD (n=6).

* *P*<0.05 and

** *P*<0.01, compared with the Sham group.

## Discussion

It has been reported that the morbidity of cognitive impairment after CPB cardiac surgeries has reached up to 20%-80% (5). CPB can result in cerebral injury, which may cause ischemia, anoxia and neuronal injury (-). This study focused on the influence of pretreatment with sufentanil on brain water content, serum S100β, brain total calcium, blood pressure, heart rate, and blood gases in CPB period.

Besides, some brain diseases could also reflect by brain water content. The brain edema is an unfavorable clinical complication resulting from a progressive increase in brain water content, often occurring secondary to various pathological conditions including cerebral infarction, hemorrhage, trauma, infection, and neoplasms (25). In encephaledema, the tectology of cerebral cortex is changed, which can be evaluated by the water content of brains (26). This study showed that pretreatment with sufentanil reduces the brain water content during CPB period indicating moderate sufentanil pretreatment can protect brain against injuries such as cerebral edema. 

Evidence has also shown that intracellular calcium overload may be the underlying mechanism of cerebral ischemia (27, 28). The neuronal activities and synaptic impairment may directly be influenced by imbalance of calcium in synapses (29). We also measured the total calcium in brain tissue to compare the intracellular calcium of rats in each group. We found that the total calcium level in the brain tissue was significantly higher in the CPB group than that of the Sham group, indicated that the total calcium in the brain tissue is correlated with cerebral injury. Modulation of the intracellular Ca^2+^ disposition and closing voltage-gated Ca^2+^ channels on presynaptic nerve terminals are the mainly molecular mechanism of opioid desensitization (30, 31). Thus, as a type of μ opiate receptor stimulant, sufentanil was speculated to suppress the overload intracellular calcium. As expected, we found that the total calcium of the brain tissue in rats with sufentanil pretreated groups, especially S1, was significantly lower than that in the CPB group, suggesting that the concentration of sufentanil pretreatment could effectively reduce the total calcium content in rat brain so that the cerebral injury could be attenuated. 

Evidence showed that the increased expression of serum S100β may be particularly relevant to the decreased cognitive function (7). S100β is a kind of neuropeptide, which was mainly generated by activated astrocytes. S100β mainly functions as nutrition to spongiocytes, promoting axon potentiation and processing information through neurotransmission at synapses. Meanwhile, it could combine with free Ca^2+^ to regulate the concentration of Ca^2+^ on the membrane. High concentration of S100β could induce neuronal apoptosis (32). Recently, researchers hypothesized that cerebral injury allows the permeation of S100β protein from intercellular fluid to cerebrospinal fluid (33, 34), and finally S100β in cerebrospinal fluid was increased and entered the blood through damaged Blood Brain Barrier (BBB) (8). Thus, the content of S100β in the cerebrospinal fluid and blood is representative to the severity of cerebral lesion as well as the degree of the BBB injury (35). In our study, we found that the level of serum S100β in S1 and S5 groups after sufentanil pretreatment was significantly lower than that of the CPB group, indicating that decreased expression of S100β may be involved in the protective effect of the sufentanil pretreatment after CPB. Also, the protective effect of the sufentanil pretreatment was more significant at the concentration of 1 µgKg^-1^.

 The values of mean arterial blood pressure is usually down-regulated during CPB (36). However, Sun *et al. *(37) indicated that there was no significant difference in the incidence of low blood pressure on sufentanil-induced cough during anesthetic induction among groups pretreated with different doses of dexmedetomidine. This phenomenon also observed in some patients with cerebral injury after sufentanil treated (38). In this study, the blood pressure are similar among groups CPB, S1, and S5 in the perioperative period of CPB, indicating that pretreatment with sufentanil play no effective role in blood pressure. 

Heart rate variability is a reliable reflection of many physiological factors modulating the normal rhythm of the heart (39). In a previous study (40), epidural analgesia treated with sufentanil is associated with fetal heart rate to be more prosperous    (41). However, in our study, no obvious difference was observed in sufentanil pretreated groups S1 and S5 compared with CPB group in the perioperative period of CPB. This result indicated that the trend of heart rate change with may correlate with different symptoms, disease or biological effects of sufentanil. In addition, pretreatment with sufentanil also showed no statistically significant change inarterial blood gases compared with CPB group in the perioperative period of CPB. Further investigation requires to find the mechanism of this effect. 

In summary, this report showed that administration of sufentanil has protective effect on cerebral injury during CPB cardiac surgeries may be through reduction of water content and total calcium in brain and S100β in serum. Pretreatment of 1 µgKg^-1^ sufentanil could be sufficient to prevent cognitive damages of brain functions under the condition of CPB and reduction in calcium overload may be a potential mechanism in this process.
